# Biological and Medical Importance of Cellular Heterogeneity Deciphered by Single-Cell RNA Sequencing

**DOI:** 10.3390/cells9081751

**Published:** 2020-07-22

**Authors:** Rishikesh Kumar Gupta, Jacek Kuznicki

**Affiliations:** 1 International Institute of Molecular and Cell Biology in Warsaw, Trojdena 4, 02-109 Warsaw Poland; jacek.kuznicki@iimcb.gov.pl; 2 Postgraduate School of Molecular Medicine, Warsaw Medical University, 61 Żwirki i Wigury St., 02-091 Warsaw, Poland

**Keywords:** scRNA-seq, transcriptomics, cell-to-cell heterogeneity, machine learning, artificial intelligence

## Abstract

The present review discusses recent progress in single-cell RNA sequencing (scRNA-seq), which can describe cellular heterogeneity in various organs, bodily fluids, and pathologies (e.g., cancer and Alzheimer’s disease). We outline scRNA-seq techniques that are suitable for investigating cellular heterogeneity that is present in cell populations with very high resolution of the transcriptomic landscape. We summarize scRNA-seq findings and applications of this technology to identify cell types, activity, and other features that are important for the function of different bodily organs. We discuss future directions for scRNA-seq techniques that can link gene expression, protein expression, cellular function, and their roles in pathology. We speculate on how the field could develop beyond its present limitations (e.g., performing scRNA-seq in situ and in vivo). Finally, we discuss the integration of machine learning and artificial intelligence with cutting-edge scRNA-seq technology, which could provide a strong basis for designing precision medicine and targeted therapy in the future.

## 1. Introduction

Multicellular organisms contain cell-type-specific combinations to form a whole-body structure. However, a particular cell type becomes privileged over other cell types because of its distinct tolerance and exposure to the same environmental challenges. Thus, classifying cell-to-cell heterogeneity is essential for understanding the ways in which development occurs and the ways in which functions are defined for specific bodily organs. Nevertheless, cell-to-cell heterogeneity and stochasticity in gene expression are inherent to all living cells and impose phenotypic and functional variations of individual cells that facilitate their adaptability to dynamic environmental conditions [1,2]. However, less is known about how to define molecular heterogeneity that is present in the cell.

Cell-cycle stages and cell types are well-understood dimensions that are routinely applied to define phenotypic cell-to-cell heterogeneity [3,4]. However, for a given phenotypic state of genetically homogeneous cells, specific responses to the same stimulus have not been sufficiently defined to understand the pathological mechanism of disease states because of a lack of understanding of several other dimensions that contribute to cell-to-cell heterogeneity. Efforts to determine the extent of cell-to-cell heterogeneity in heterogeneous cell populations in an unbiased manner have invigorated the emergence of new analytical standards that integrate innovative platforms. Single-cell transcriptomics is one such approach [5]. Almost a decade has passed since single-cell RNA sequencing (scRNA-seq) technology was developed. It has since been applied to various research domains to answer fundamental biological questions [5,6,7,8,9,10]. The dynamics of gene expression have changed significantly over time [11], and scRNA-seq has allowed quantification of the expression of individual genes across cells [12].

It has been a decade since scRNA-seq technology was developed and evolved to be more advanced. In this article, we describe the cellular heterogeneities that have been identified in various organs, body fluids, and pathologies such as cancer and neurological disorders. We outline the heterogeneity discovered by scRNA-seq techniques, which could be used for the identification of stem cells, the discovery of new biomarkers, and the state of transcriptomic trajectories in response to treatment within a cell population over time. In addition, along with scRNA-seq techniques, we highlight the possible implementation of machine learning and artificial intelligence (AI), which could be evolutionary in personalized and precision medicine. The present review provides insight for the beginner in scRNA-seq research.

## 2. Platforms Presently Used for Single-Cell RNA Sequencing (scRNA-seq)

**Figure 1:**
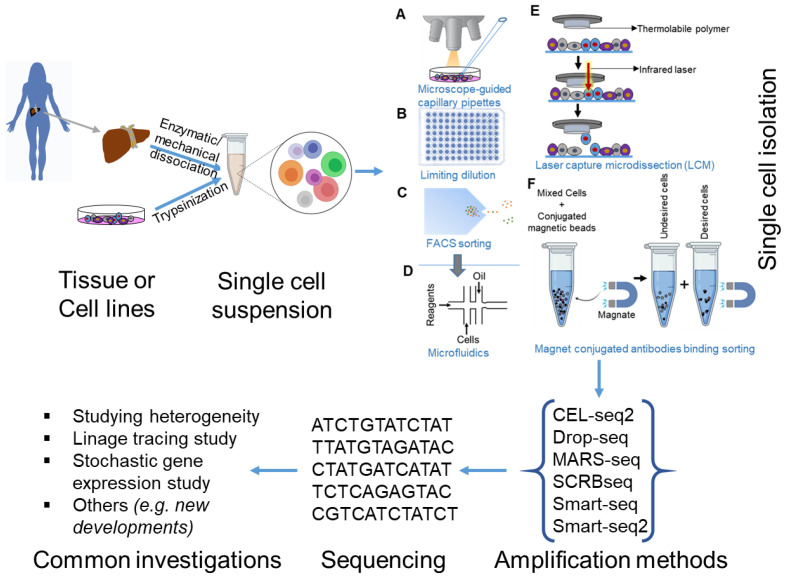
Overview of single-cell RNA sequencing (scRNA-seq) methodology. Single-cell RNA sequencing technology is used to explore transcriptomic profiles of single cells that are isolated from cell lines, organisms, or tissue/blood samples of clinical material. Massive datasets can be generated and analyzed by a specific algorithm that allows the discernment of cell-to-cell heterogeneity, lineage tracing, and stochastic gene expression at the single-cell level.

The development of new effective protocols for scRNA-seq is currently a very active area of research. The primary steps of scRNA-seq protocol are the following: sample preparation and single cell isolation by many approaches such as limiting dilution, microscope-guided capillary pipettes, laser capture microdissection (LCM), and magnet conjugated antibody binding sorting; the digestion of solid tissue by mechanical or enzymatic methods and single-cell capture by fluorescence-activated cell sorting (FACS) or a microfluidic device and cell lysis; scRNA-seq (reverse transcription, amplification, library preparation (based on type of coverage) on 3′- or 5′-based ends or full-length transcripts and sequencing); data processing (cell-specific reads and alignment with a reference genome); and data analysis and interpretation (marker identification and cell clustering into potential cell types and states) (Figure 1). FACS in multiwall plates, microfluidic channels, and a combination of both are frequently used to isolate the single cell. Moreover, microfluidics comprise the formation of droplets that contain reagents and beads which are combined with unique molecular identifiers, also known as a tag-based (also known as unique molecular identifiers (UMIs)) protocol that aids quantification [13]. However, only ~11 to 65% of the cells from an original sample are captured by a microfluidic platform. Thus, significant amounts of cell inputs are needed, and this protocol can miss rare cell types. Alternative approaches for scRNA-seq have been recently described, such as CEL-seq2, Drop-seq, MARS-seq, SCRBseq, Smart-seq, Smart-seq2, and Patch-seq (combining electrophysiology with transcriptomics over a large variety of cells) [14,15,16,17]. Understanding the significance of each platform gives us the opportunity to choose the platform based on our aim. CEL-seq2, Drop-seq, MARS-seq, and SCRB-seq are used to quantify the mRNA levels with less amplification noise. MARS-seq, SCRB-seq, and Smart-seq2 platforms are efficient when analyzing fewer cells. In addition, it is worth highlighting that Smart-seq2 detects the most genes per cell, however, across cells, Drop-seq is the most cost-efficient when the aim is to quantify a large number of cells.

## 3. Analyze the scRNA-seq Data

Along with the technological advancement to perform the comprehensive scRNA-seq experiment, analyzing the high throughput data from hundreds of thousands of cells to extract the biologically relevant information is rather challenging. Demultiplexing; alignment with the reference genome, barcode, and UMI filtering; marking duplicates; and filtering cells are the series of events to analyze scRNA-seq data [18]. This allows us to profile the subpopulations or types of cells from a pool of heterogeneous mixtures of cells. While analyzing the scRNA-seq data, the main focus is to find out the number of distinct molecules that can be captured from each cell and based on this information define the cellular heterogeneity. We can cluster a heterogeneous cell population based on the gene expression patterns, and by identifying the specific gene markers for a cluster, we can identify the cell types. We could possibly identify an unprecedented cell cluster, which could be a new cell type that was not identified previously. Currently, scRNA-seq data techniques can identify the phase of the cell cycle of a specific cell, which provides great details in the case of a tumor biopsy for deciphering the heterogeneity of the cells and evaluating the tumor microenvironment, understanding metastasis, as well as monitoring and predicting the response to therapy [19,20,21,22,23,24]. A new approach called effective and expressed nucleotide variations (eeSNVs) associated with gene expression has been developed to identify the features in a tumor subpopulation identification from scRNA-seq data [25,26]. The authors have suggested that eeSNVs achieve better accuracies for identifying subpopulations. They have also suggested that their analytical technique was capable of analyzing coupled DNA-seq and RNA-seq data from the same single cells, demonstrating its value in subpopulation identification and linkage of genotype–phenotype relationships. Furthermore, advance analysis such as the maximum parsimony analysis [27,28] and spatial mapping analysis [29,30,31] provide temporal dynamics and spatial position of a heterogeneous mixture of cells in various states.

## 4. Advantage of scRNA-seq over Regular RNA-seq

A typical RNA-seq experiment informs about the average gene expression across a cell population. The level of mRNA is estimated across all cells of each subpopulation. However, this approach can miss the fact that individual cells could have patterns of expression that are very different from the average expression. In any tissue or organ (e.g., blood, brain, and cancer biopsies), the cell population is heterogeneous. Such cell-to-cell variability can have important biological implications that can be deciphered only by using scRNA-seq data. The data provide information about the degree of significance and meaning of a specific proportion of cells that have a specific gene expression pattern. This knowledge is essential for a better understanding of the function of tissues and organs and associated pathologies. Defining cellular heterogeneity in diseased tissue allows the identification of precise drug targets and possibly the development of new therapeutic approaches. scRNA-seq data has been widely used across all biological disciplines, including developmental biology [32], neurobiology [33], cancer biology [7], and autoimmune and infectious diseases [5,9]. For example, during development, progenitor cells undergo dynamic cellular and molecular changes to generate diverse populations of different cell types. Such transcriptomic heterogeneity can be effectively identified by using scRNA-seq data. This technology has also substantially contributed to our understanding of the development of different mammalian organs and tissues and the development of multicellular organisms [4,10,11,34,35,36,37,38,39,40,41,42,43,44]. The possible applications of scRNA-seq data are wide-ranging, including the identification of stem cells, the discovery of new biomarkers, and the detection of transcriptomic trajectories within the cell population in response to medical treatments over time. scRNA-seq technology has become promising for successfully developing individually designed medicinal treatments for cancer and other diseases.

## 5. Cellular Heterogeneity Deciphered by scRNA-seq in the Cardiovascular System

According to the WHO, cardiovascular diseases (CVDs) are the most prominent cause of death globally [45]. The WHO broadly defines CVDs as a group of disorders of the heart and blood vessels, which include coronary heart disease, cerebrovascular disease, peripheral arterial disease, rheumatic heart disease, congenital heart disease, and deep vein thrombosis and pulmonary embolism [45]. Various approaches are available to treat heart diseases such as a change in lifestyle, stress management, certain medications [46], and surgical intervention. However, the remarkable heterogeneity in cell types and functional states is the major challenge in understanding cardiac biology and disease. scRNA-seq data provide the prospect of profiling the transcriptional landscape of cardiac cells and revealing the significant heterogeneity in both healthy and disease states to identify clinically crucial diagnostic biomarkers and a rich resource for studying cardiac biology. A recent scRNA-seq study on human induced pluripotent stem cell-derived cardiomyocytes (hiPSC-CMs) provided a powerful understanding of in vitro cellular heterogeneity (Table 1) [47]. From the scRNA-seq data, the authors identified multiple enriched subpopulations for *TBX5*, *NR2F2*, *HEY2*, *ISL1*, *JARID2*, or *HOPX* transcription factors in differentiating hiPSC-CMs. In cell lineages forming the heart, the highest cell-to-cell heterogeneity appears during embryonic development, and multipotent cells undergo a series of differentiation to reach the ultimate fate. scRNA-seq data on mouse cardiac progenitor cells (CPCs) from E7.5 to E9.5 have shown eight different cardiac subpopulations and provided an understanding of transcriptional and epigenetic regulations during cardiac progenitor cell fate decisions at a single-cell resolution [48]. Another study using scRNA-seq data from mouse E10.5 stage cardiac cells from heart chambers (five anatomic zones, i.e., left ventricle, left septum, right septum, right ventricle, and atrial ventricular canal) identified 12 subpopulations and revealed that the cell cycle was a major determinant of expression variation in all cardiac cell types which selectively regulate cardiac chamber growth during development, and provided a deeper understanding of the pathogenesis of congenital heart disease [49]. Apart from CMs, many other cell types are there, which play a role in heart repair, regeneration, and disease. A recent article on scRNA-seq data from murine hearts on days three and seven post sham or myocardial infarction (MI) for >30,000 single cells identified >30 populations which broadly represented nine cell lineages and identified novel myofibroblast subtypes [50]. This analysis provided a deeper analysis of cardiac homeostasis, inflammation, fibrosis, repair, and regeneration. A scRNA-seq data profile of 21,422 cells (CMs and NCMs) from healthy, failed, and partially recovered (left ventricular assist device treatment) adult human hearts revealed an inter- and intra-compartmental CM heterogeneity and provided insights into the cell-type-targeted intervention of heart diseases [51]. A scRNA-seq study on circulating immune cells in patients with heart failure has shown three subpopulations of monocytes as compared with healthy subjects. This study also indicated that scRNA-seq could be useful as a prognostic tool or for guiding anti-inflammatory therapies associated with cardiovascular disease [52].

## 6. Profiling Heterogeneous Cell Populations from Different Tumors

Dissecting cell-to-cell heterogeneity is extremely important for understanding tumor initiation, progression, metastasis, and therapeutic responses. Many attempts have been made using next-generation sequencing and microarray technology [53,54,55,56]. In cancer, the population of cells has highly diverse morphology, phenotypic profiles, genetics, metabolism, motility, proliferation, and metastatic potential, which poses significant challenges for designing effective treatment strategies [57,58,59]. A scRNA-seq analysis can discriminate functionally healthy cells from cancer cells at various developmental stages of tumors to allow more precise prognoses and the determination of sensitivity to different drugs for developing the most effective treatment strategies [23]. A scRNA-seq study of syngeneic tumors in a mouse model identified potential ligand–receptor interactions between cells within the tumor microenvironment, suggesting that specific ligand-receptor pairs could be potential biomarkers or drug targets [60].

Breast cancer in women is the most prevalent tumor worldwide. The results of a scRNA-seq analysis of 11 triple-negative breast cancer patients showed a high level of intratumoral heterogeneity in tumor cells (Table 2) [61]. A scRNA-seq analysis of mesenchymal cells from a genetically engineered mouse model of breast cancer was performed. The authors reported compelling evidence of three spatially and functionally distinct subpopulations of breast cancer-associated fibroblasts [62,63]. Another study performed scRNA-seq of estrogen receptor α-positive breast cancer cells following 17β-estradiol stimulation and reconstructed the dynamic estrogen-responsive transcriptional network from discrete-time points into a pseudotemporal continuum [64]. They provided evidence that estrogen signaling promoted breast cancer cell survival and growth by mediating a metabolic switch. Hydro-seq, a recent high-throughput parallel scRNA-seq study, captured circulating tumor cells with high efficiency and low contamination [19]. The authors performed scRNA-seq of 666 circulating tumor cells on samples from 21 breast cancer patients and identified drug targets for hormone therapy. This technology allowed the identification of biomarkers with high fidelity based on the transcriptomic heterogeneity of rare cells that were present in the blood.

The incidence of liver diseases is rising, but only limited treatment options are available. A recent scRNA-seq study provided a glimpse of B cell and plasma cell subsets in the human spleen and liver and revealed the heterogeneity of liver-resident immune cells (LrICs) [69]. From nine human donors, a single-cell transcriptomic atlas of 10,372 liver cells from healthy liver tissue has been constructed, revealing cellular heterogeneity [70]. The authors identified cell types that could be defined by the expression of marker genes, including hepatocytes, bile duct cells (cholangiocytes), liver sinusoidal endothelial cells, macrovascular endothelial cells, hepatic stellate cells, myofibroblasts, Kupffer cells, and immune cells. A scRNA-seq study of hepatocellular carcinoma revealed the distinct functional composition of T-cells. The study performed scRNA-seq of 5063 single T-cells that were isolated from peripheral blood, tumor tissue, and adjacent healthy tissue which were donated from hepatocellular carcinoma patients. Eleven subpopulations of T-cells were identified based on their molecular and functional properties [65].

Pancreatic cancer cell and β-cell degradation leads to multiorgan damage, including nephropathy, retinopathy, and enteropathy. scRNA-seq analyses of the human pancreas has allowed parallel identification of cell population subtypes and disease-associated alterations of gene expression. A recent scRNA-seq study of pancreatic ductal adenocarcinoma (PDAC) patient donor tissue and healthy pancreatic tissue compiled a transcriptomic atlas of 57,530 pancreatic cells and revealed a connection between the intrinsic transcriptional state of the tumor and T-cell activation [66]. This suggests the possibility of identifying biomarkers for anticancer treatment against T-cell activation as targeted immunotherapy [66,71]. The authors identified heterogeneous malignant subtypes that were composed of several subpopulations with differential proliferative and migratory potential, including two ductal subtypes. The ductal subtype (type 2) was associated with an inactive state of tumor infiltration T-cells, which could be a unique antitumor immune response. Another scRNA-seq analysis generated a multiscale transcriptomics map that was based on patient donor pancreatic tissue, a pancreatic tumor mouse model, and a human cell line that originated from the tumor [72]. This study found that the nuclear hormone receptor retinoic-acid-receptor-related orphan receptor γ was upregulated during pancreatic cancer progression. This could be another therapeutic target for reducing tumor burden and improving survival.

Neuroblastoma is the third most common cancer in children [73]. A scRNA-seq analysis revealed the following three heterogeneous cell types in the neuroblastoma cell lines: (i) sympathetic noradrenergic cells (expressing *PHOX2B*, *HAND2*, and *GATA3* transcription factors); (ii) neural crest cells (expressing the *AP-1* transcription factor); and (iii) a mixed type [67]. This study revealed neuroblastoma cell heterogeneity and provided insights into developing novel neuroblastoma treatment strategies.

Acute myeloid leukemia (AML) is a cancer of the myeloid line of blood cells, which most commonly occurs in older adults, and males are affected more often than females [74]. It is well known that AML is a very heterogeneous disease, as different cell types contribute to AML progression. However, it remains elusive with respect to what the sources are to raise the heterogeneity and how to classify the microenvironmental cellular heterogeneity in the case of AML. A recent scRNA-seq analytical technique called PhenoGraph has been developed to simultaneously profile both surface and signaling features from millions of pediatric leukemic cells. This method provides a framework to discover other features of molecular cell biology to find out the associated mechanistic or clinical outcomes [75]. Additionally, a recent scRNA-seq analysis of 38,410 cells from 40 bone marrow aspirates, provided an atlas of AML cell states, regulators, and markers at single-cell resolution [68]. The data showed that differentiated monocyte-like AML cells expressed diverse immunomodulatory genes. The authors were able to identify six types of malignant cells and concluded that these findings could be useful in implications for precision medicine and immune therapies. Another very recent research article has identified that in AML samples, transcriptional heterogeneity arises from multiple sources, including the differentiation states of normal and tumor cells, cell cycle states, and mutations [76]. The authors also claimed that nongenetic heterogeneity arose as a consequence of stochastic gene expression or other external and internal perturbations.

In recent years, scRNA-seq has been extensively used to understand the intratumoral genetic landscape in glioblastoma, medulloblastomas, kidney cancer, colorectal cancer, and other types of cancers [20,21,24,59,77,78,79,80,81,82,83,84,85,86,87,88]. A recent scRNA-seq study provided a blueprint for glioblastoma and found that malignant cells exist in four primary cellular states, and the relative frequency of cells in each state varied according to the tumor microenvironment [89]. A scRNA-seq study of approximately 2400 cells from human blood identified six new dendritic cells (DC), four monocytes, and one progenitor cell subtype [90]. The authors also found a new DC subpopulation that shared properties with plasmacytoid DCs (pDCs) and activated T-cells. This revised taxonomy enabled more accurate functional and developmental analyses and immune monitoring in health and disease.

## 7. Heterogeneity in Neurons under Normal and Pathological Conditions

scRNA-seq has been used to identify genes that are involved in neural communication, which broadens our knowledge about brain function and could provide new strategies to understand the mechanisms of neurological disorders. Microglia play a vital role in neurodevelopment and neurodegeneration, such as Alzheimer’s disease [91] and Parkinson’s disease [92], and have been extensively studied with regard to their role in demyelination and the induction of different cellular kinetics [93]. The heterogeneity of microglia from healthy and neuropathologic tissues has been explored using scRNA-seq (Table 3) [94]. This study found 13 distinct, time- and region-dependent clusters of microglia in healthy human brains and brains from patients with multiple sclerosis. Ten of these 13 clusters were present during development, two were present during demyelination and remyelination, and one was present during neurodegeneration. Disease-associated subtypes differed between toxic demyelination and neurodegeneration. The transcriptional profiles of subtypes of microglia depended on the context, which could be significant for identifying new drug targets.

Alzheimer’s disease is one of the most challenging neurological disorders. A recent scRNA-seq study of 80,660 cells from the prefrontal cortex in 48 individuals with varying degrees of Alzheimer’s disease pathology found six known major brain cell types and 40 transcriptionally distinct cell subpopulations [95]. The authors also found that each major cell type exhibited a different gene expression pattern in Alzheimer’s disease pathology and demonstrated links between this response and Alzheimer’s disease risk genes that were identified by genome-wide association studies. Such a response was also shown to be sex-specific, especially in neurons and oligodendrocytes.

Parkinson’s disease results from the death of dopaminergic neurons in the substantia nigra and is the second most common neurodegenerative disorder after Alzheimer’s disease [96]. To understand the genetic complexity of dopaminergic neurons in Parkinson’s disease, a scRNA-seq analysis was performed in a 1-methyl-4-phenyl-1,2,3,6-tetrahydropyridine (MPTP) mouse model of Parkinson’s disease [97]. The authors identified multiple distinct dopamine neuron subtypes, each with a heterogeneous expression pattern of marker transcription factors, channels, receptors, dopamine-related genes, neuropeptides, and secretory factors. Another recent scRNA-seq study on the iPSC-derived motor neurons (MN) showed 14 cell clusters where a significant proportion of iPSCs-MNs were identified as a heterogeneous population of neural progenitor cells (NPCs), interneuron (Ins), MNs, and glial cells based on the expression of the specific genes. This elegant scRNA-seq study of iPSCs-MNs provided potential evidence to improve our understanding and lead to improved iPSC-based applications to study motor neurons related to disease in humans [98].

**Table 3:** Summary of main results from selected scRNA-seq studies in neurons under normal and pathological conditions.

Source of Cells Assayed	Seq. Method	Number of Cells	Key Results	Ref.
Brain tissue from healthy human and patients with multiple sclerosis	Cel-seq2	-	Found 13 distinct and time- and region-dependent clusters of microglia	[94]
Brain tissues from patients with Alzheimer’s disease pathology	Drop-seq	80,660	Six known major brain cell types and 40 transcriptionally distinct cell subpopulations	[95]
Dopaminergic neurons from MPTP mouse model	Smart-seq2	-	Multiple distinct dopamine neuron subtypes	[97]
Human iPSC-derived spinal motor neurons	-	5900	14 cell-clusters a heterogeneous population of neural progenitor cells (NPCs), interneuron (Ins), MNs and glial cells	[98]
Mice brain tissue	Drop-seq	6232	Diverse hippocampal cell types plays a specific role in the pathology of mild TBI	[99]
Mouse striatum cells	Smart-seq2	1208	10 heterogeneous striatal cell types	[100]
Mouse hypothalamic cells	-	31,000	70 different neuronal clusters	[31]
Mouse visual cortex cells	nDrop-seq	114,601	Eight different cell types: excitatory neurons, inhibitory neurons, oligodendrocytes, and oligodendrocyte precursor cells, astrocytes, endothelial and smooth muscle cells, pericytes, microglia, and macrophages	[101]
Olfactory epithelial tissue	-	51,246	38 heterogeneous cellular clusters	[102]
Zebrafish larvae brain cells	Smart-seq2	4365	18 distinct habenular neuronal types	[103]
Drosophila brain cells	Cel-seq2 and SMART-seq2	157,000	87 initial cell subclusters from different transcriptional states	[104]

A recent scRNA-seq study further revealed how traumatic brain injury (TBI) could impact diverse hippocampal cell types, changes in the proportion of cells, and shifts in global transcriptome patterns [99]. In a mouse model of mild TBI, significantly more ependymal cells were found as compared with sham samples. Mild TBI resulted in significant changes in the transcriptome of many hippocampal cell types, especially in the dentate gyrus granule cells. This indicated that each cell type played a specific role in the pathology of mild TBI. Two previously undefined cell populations in the hippocampus were also identified by scRNA-seq [99].

The striatum is a cluster of neurons in the subcortical basal ganglia of the forebrain medium spiny neurons (MSNs) (~95% of the total neuronal population of the human striatum), cholinergic interneurons, and many types of GABAergic interneurons. In the healthy brain, the striatum receives glutamatergic and dopaminergic inputs from different sources and coordinates multiple aspects of motor action, intellectual ability, and cognition [105,106,107,108]. Any damage to the striatal cells leads to many neuropsychiatric disorders such as Parkinson’s and Huntington’s disease, schizophrenia, obsessive-compulsive disorder, addiction, and autism [109,110,111]. A scRNA-seq study of the mouse striatum found 10 heterogeneous striatal cell types among 1208 cells that were analyzed [100]. The authors also found that two discrete types of medium spiny neurons could be further divided into different subtypes based on specific transcriptomes. They also identified cell type-specific transcription factors that could mechanistically explain the maintenance of discrete cell type identities, which gave a clue of the emergence of functional diversity within a complex tissue from a small number of discrete cell types. Another scRNA-seq study in combination with patch-clamp techniques of striatal cells found the following seven distinct interneuron types: six γ-aminobutyric acid (GABA)ergic and one cholinergic [17]. Using patch-clamp techniques, the authors identified the neuronal circuit integration based on their spiking pattern, which showed considerable electrophysiological heterogeneity within these groups of neurons. These findings widen our knowledge of the physiological properties and synaptic connectivity to understand how synaptic connections are formed and maintained in the brain.

The hypothalamic preoptic region in humans is a delicate and complex neurovascular structure that controls essential social behaviors and homeostatic functions. A scRNA-seq atlas of the mouse hypothalamic preoptic region showed approximately 70 different neuronal clusters based on molecular annotation and spatial resolution [31]. Some of these cells were not previously known. In the adult mouse hypothalamus, 45 transcriptionally distinct cell subtypes were identified, including 11 non-neuronal types and 34 neuronal types. Among the latter type, 15 were glutamatergic, 18 were GABAergic, and one was histaminergic, with distinct transcriptional signatures [112]. The authors found that food deprivation affected the transcriptome of neuronal cells differently. The differentially expressed genes were mostly observed in a few specific glutamate and GABA clusters.

The visual cortex in the brain processes visual information and shapes neuronal plasticity through an activity-dependent transcriptional response. scRNA-seq of the mouse visual cortex was applied to provide a comprehensive understanding of transcriptional changes that occur across cell types [101]. The authors analyzed 114,601 cells and identified the following eight different cell types: excitatory neurons, inhibitory neurons, oligodendrocytes, and oligodendrocyte precursor cells, astrocytes, endothelial and smooth muscle cells, pericytes, microglia, and macrophages. Surprisingly, light-responsive genes were detected in both neuronal and non-neuronal cell types. Among 611 stimulus-sensitive genes, 362 represented the early response.

In the mouse olfactory bulb, scRNA-seq uncovered markers of cell types in adult-born neurons. The authors identified 38 heterogeneous cellular clusters (sixteen neuronal cell clusters, three astrocytic cell clusters, five olfactory ensheathing cell-based clusters, six hematopoietic cell clusters, four blood vessel-based cluster, one oligodendrocyte precursor-based cluster, one myelinating oligodendrocyte-based cluster, and two mesenchymal clusters) [102]. Adult-born neurons were shown to use at least two different molecular pathways to mature, and they differentially respond to olfactory experience. These findings provided a framework for studying cell type-specific functions and circuit integration in the mammalian brain.

Mice, rats, and other vertebrate animals have been used to elucidate the mechanisms of human diseases. One notable study established a scRNA-seq protocol for neurons in larval and adult-stage zebrafish and identified 18 distinct habenular neuronal types, although the cell types retained the same identity from the larval-stage to adult-stage habenula, despite brain growth and functional maturation [103]. However, neuronal types in the zebrafish habenula have high diversity with regard to neuronal excitability, neurotransmission, and peptidergic signaling [103]. A recent scRNA-seq study of an adult zebrafish model of the Alzheimer’s disease brain showed the existence of heterogeneous and spatially organized neural stem cells in the telencephalon. They found that different stem cell populations reacted differently to amyloid β-42 toxicity [113].

Changes in the transcriptomic profiles of brain cells during aging were analyzed in *Drosophila* using scRNA-seq [104]. The authors created a scRNA-seq atlas of all adult *D. melanogaster* brain cells across the lifespan and identified 87 initial cell subclusters. They concluded that aging mechanisms are cell type-specific, and cell state changes can be identified by gene expression markers, gene signatures, transcription factor expression, or regulons. Moreover, glial cells showed a clear aging trajectory in their transcriptional profile, while an exponential decline in gene expression correlated with neuronal shrinking. Another scRNA-seq study on drosophila olfactory projection neurons classified neuronal subtypes defined by transcriptomic identity, corresponding with the anatomical location and physiological activity [114]. This study concluded that transcription factors and cell surface molecules could be used as key determinants of cell fate.

## 8. Cell-to-Cell Heterogeneity during Development

Hans Driesch’s sea urchin experiment in 1892 provided many insights into the fact that any single cell in the early embryo was capable of forming developed larva, concluding that the fate of a cell was not predetermined but rather reflected the progressive emergence of complexity [115]. Driesch also suggested that the emergence and diversification of cell types actively lead to animal evolution. Cell-to-cell heterogeneity is an inherent property that determines phenotypic differences within species and among individuals. Therefore, cell-to-cell heterogeneity cannot be ignored when interpreting and analyzing the process of animal development. A scRNA-seq study of *Caenorhabditis elegans* nematode larva development identified 18 non-neuronal cell types and many neuronal cell types that could be grouped into either ten broad classes or 40 fine-grained clusters [41]. A scRNA-seq gene expression analysis of *Schmidtea mediterranea* (flatworms) molecularly characterized 37 cell types, including 23 terminally differentiated cell types, progenitor cells, and stem cell clusters [4]. An integrative whole-organism scRNA-seq analysis of *Nematostella vectensis* cnidarian larvae and adult cell type revealed cell type complexity [42]. The authors uncovered eight broad classes of cells, each class comprising different subtypes that were defined by the expression of multiple specific markers. This study provided insights into the evolution of animal cell-specific genomic regulation. However, to reveal the dynamic features of cell differentiation pathways, a time-series scRNA-seq analysis approach was applied in embryos of the western clawed frog *Xenopus tropicalis* [11]. The authors created developmental cell trajectories and identified 259 cell states that were composed of 136,966 cells and spread over 10 time points, identifying conserved and divergent features of vertebrates in early developmental stages. Another scRNA-seq study of more than 90,000 cells from the onset of gastrulation of the swimming tadpole *Ciona intestinalis* defined comprehensive transcriptome trajectories, regulatory cascades, and gene networks for over 60 cell types (including nearly 40 neuronal subtypes) [44]. On the basis of the dual properties of the *Ciona* notochord and expansion of the vertebrate forebrain, this study also provided an understanding of the evolutionary transition between invertebrates and vertebrates. It also offered an unprecedented opportunity to trace the evolutionary origins of every cell, tissue, and organ.

## 9. Application of Machine Learning to Assess Cellular Heterogeneity from scRNA-seq Data

Artificial intelligence and machine learning are making their way into almost every domain of research. These technologies refer to the use of data by machines to simulate human intelligence processes, including reasoning, learning, and self-correction. The rapid development of computation and machine learning, combined with the generation of massive datasets, has fueled the development of new technologies in machine intelligence for the scRNA-seq data analysis and prediction of cell fate. These techniques can identify appropriately supervised deep learning architectures and training algorithms for scRNA-seq data and be used to explore generative adversarial network methods for the estimation of high-dimensional data distribution in the single-cell gene expression space [116]. These approaches can be used to develop new technologies and address unresolved issues of single-cell genomics, such as the pseudotemporal ordering of single-cell data, the clustering of data, investigations of representations, transfer learning, and unsupervised feature discovery. A recent study reported the unbiased identification of genes that were able to distinguish subtypes using a machine-learning algorithm called iterative clustering for identifying markers [114]. This algorithm works recursively, examines finer-grained subpopulations, and is capable of detecting the transcriptomics landscape that can distinguish small subpopulations. scRNA-seq technology produces an immense amount of data, and biased analysis, technical noise, and stochastic behavior of the data could lead to conjecture instead of finding out the correct conclusion. AI and machine learning could provide the most promising solution to overcome these problems. It is worth mentioning that the integration of scRNA-seq technology with AI and machine learning would provide a better understanding and could be timely and much more efficient. Single-cell variational inference (scVI) is a recent development in this direction, which is a ready-to-use scalable framework to analyze scRNA-seq data [117]. scVI takes raw count data as input, uses stochastic optimization, and deep neural networks learn a cell-specific scaling factor as a hidden variable, intending to maximize the likelihood of the data, and then perform fundamental analysis with high accuracy, such as batch correction, visualization, clustering, and differential expression; however, this algorithm does not provide an analysis of lineage inference and cell-state annotation. Another machine learning-based model has been developed, called scGen, combining variational autoencoders and latent space vector arithmetic which has the capacity to model perturbation and infection response accurately in cells across cell types, studies, and species which would increase our understanding in the screening of perturbation response in the context of disease and drug treatment [118]. Moreover, recently, by combining deep autoencoders with a Bayesian model, SAVER-X, a more robust framework, has been developed which trains a deep neural network across a range of study designs and applies this model to new data to obtain the shared biological information across different data [119].

## 10. Frontiers in scRNA-seq Application

**Figure 2:**
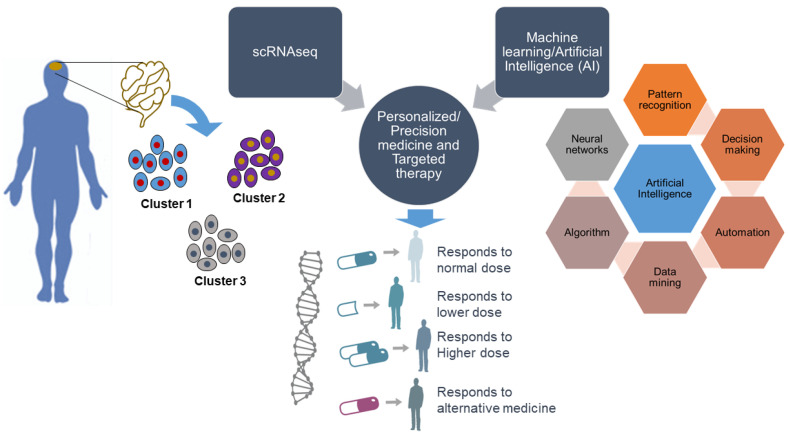
Overview of scRNA-seq technology integrated into machine learning and artificial intelligence. Single-cell RNA sequencing technology allows quantification of the expression of each gene in a cell. The integration of scRNA-seq technology with artificial intelligence enables the identification of cell-to-cell heterogeneity more accurately and would open a window to better applications.

scRNA-seq generates transcriptomic profiles of individual cells and unlocks their identity. Thus, defining heterogeneous gene expression at the single-cell level can provide a better understanding of the specificity of any specific disease to discover new genes as drug targets. More knowledge of gene expression patterns opens a significant window for the application of scRNA-seq to personalized and precision medicine and targeted therapy. Together with scRNA-seq, the implementation of machine learning and artificial intelligence, referred to as intelligent single-cell RNA sequencing (iscRNA-seq), could be more evolutionary in personalized and precision medicine (Figure 2). iscRNA-seq is a machine intelligence technology that performs real-time intelligent on-demand scRNA-seq with high throughput. It enables the identification of high-content cell clusters with unique spatial gene expression traits. iscRNA-seq serves as an integral part of holistic cell type-specific transcriptome analysis. iscRNA-seq is based on the seamless integration of high-throughput scRNA-seq and deep learning on a hybrid software-hardware data management infrastructure, enabling real-time automated operations for data acquisition, data processing, and intelligent decision making, and accelerating more precise molecular network pathway construction. This provides future opportunities for machine learning to enable the discovery of unknown gene functions and uncover the relationships between gene expression patterns and associated phenotypes. One recent advance is called single-cell, thiol (SH)-linked alkylation of RNA for metabolic labeling sequencing (scSLAM-seq), which can differentiate between new and old RNA for thousands of genes per single cell and can explain differences in transcriptional stochasticity at the single-cell level [120]. This kind of advancement will help identify the dynamics of gene expression at the single-cell level.

The present technology allows the identification of epigenetic modifications of RNA in single cells and the proteome in specific organelles, such as mitochondria [121]. Future technologies will likely draw a link between gene expression patterns that are established by scRNA-seq and proteins in each cell and its components. The application of machine learning should help further our understanding of the relationships among the regulation of gene expression by non-coding RNA, the level of coding mRNA, and their involvement in the development of pathologies. One can also expect that the field will progress beyond its present limitations and allow scRNA-seq and single-cell proteome analyses [122] in situ and in vivo, as described in recent developments of DNA microscopy [123]. In this technique, the use of DNA “bar codes” can pinpoint a molecule’s relative spatial positions within a sample and offers an entirely new way to build a picture of the cell [29,123]. The next advance in scRNA-seq could be “RNA microscopy,” which would reveal the real-time dynamics of gene expression in a living cell. The integration of RNA microscopy with artificial intelligence would open a new era in precision medicine. These new developments would significantly increase our knowledge about the function of cells, tissues, and organs, resulting in better diagnoses, drug designs, and treatments that are tailored to the needs of individual patients.

## 11. Future Directions for Single-Cell Technologies

Reports on scRNA-seq technology are growing at an extraordinary rate, but substantial work still needs to be done to validate genes that are relevant to disease. To achieve this goal, the application of scRNA-seq for a particular disease to define a gene-expression profile at the single-cell level is essential. Non-coding RNA and epigenetic modifications play a major role in altering a cell’s state, which needs to be considered when analyzing scRNA-seq data. Because diseases are associated with many secondary pathologies, cellular heterogeneity at the protein level also needs to be considered to clarify the interplay between cell type and functional state.

## Figures and Tables

**Table 1:** Summary table of main results from selected scRNA-seq studies classifying cell types in the cardiovascular system.

Source of Cells Assayed	Seq. Method	Number of Cells	Key Results	Ref.
Human induced pluripotent stem cell-derived cardiomyocytes (hiPSC-CMs)	ChIP-seq	10,376	Identified multiple subpopulations enriched for TBX5, NR2F2, HEY2, ISL1, JARID2, or HOPX transcription factors	[47]
Mouse cardiac progenitor cells (CPCs)	SMART-seq2	-	Eight different cardiac subpopulations	[48]
Mouse E10.5 stage cardiac cells from heart chambers	Ht-seq	10,000	Identified 12 subpopulations and reviled that the cell cycle is a major determinant of expression variation	[49]
Murine hearts cells	SMART-Seq	>30,000	Identified >30 populations which broadly represent nine cell lineages	[50]
Adult human hearts cardiomyocytes (CMs) and non-CMs (NCMs)	Drop-seq	21,422	CMs (atrial and ventricular) each formed five distinct subclusters.	[32]
			NCMs (ECs, FBs, MPs and SMCs) into 14 (4, 3, 3, and 4) subclusters	
Circulating immune cells	-	181,712	Circulating immune cells in patients with heart failure has shown the three subpopulations of monocytes as compared with healthy subjects	[52]

**Table 2:** Summary table of main results from selected scRNA-seq studies classifying cell types from different tumors.

Source of Cells Assayed	Seq. Method	Number of Cells	Key Results	Ref.
Circulating tumor cells (CTCs) from breast cancer patient	Hydro-seq	666	Identified the cells based on expression of ER, PR, and HER2 which could act as biomarkers	[19]
Human renal tumors and normal tissue from fetal, pediatric, and adult kidneys	-	72,501	Identified total 110 subtypes of cells	[21]
Primary glioblastomas cells from patients	SMART-seq	430	Cells from each tumor patients demonstrate higher overall intratumoral coherence, and several cells showed positive correlations with cells from other tumors	[20]
Breast cancer cells from patients	Tru-seq	515	Identified 11 clusters, mixture of tumor cells and immune cells	[61]
T-cells that were isolated from peripheral blood, tumor tissue, and adjacent healthy tissue from hepatocellular carcinoma patients	Smart-seq2	5063	Eleven subpopulations of T-cells were identified based on their molecular and functional properties	[65]
Primary PDAC tumors and control pancreases	-	57,530	Identified 10 main clusters (type 1 ductal, type 2 ductal, acinar, endocrine, endothelial, fibroblast, stellate, macrophage, and T and B cells)	[66]
Neuroblastoma cells from donor patients and cell lines	ChIP-seq	-	Three heterogeneous cell types in neuroblastoma cell lines: (i) sympathetic noradrenergic cells, (ii) neural crest cells, and (iii) a mixed type	[67]
Bone marrow aspirates from AML patients and healthy donors	Seq-Well	38,410	Differentiated monocyte-like AML cells expressed diverse immunomodulatory genes	[68]
